# Primary aldosteronism: Pathophysiological mechanisms of cell death and proliferation

**DOI:** 10.3389/fendo.2022.934326

**Published:** 2022-08-08

**Authors:** Martina Tetti, Siyuan Gong, Franco Veglio, Martin Reincke, Tracy Ann Williams

**Affiliations:** ^1^ Medizinische Klinik und Poliklinik IV, Klinikum der Universität München, Ludwig-Maximilians-Universität (LMU) München, München, Germany; ^2^ Division of Internal Medicine and Hypertension, Department of Medical Sciences, University of Turin, Turin, Italy

**Keywords:** adrenal adenoma, adrenal gland, aldosterone, cell death, endocrine hypertension, ferroptosis, hyperaldosteronism, proliferation

## Abstract

Primary aldosteronism is the most common surgically curable form of hypertension. The sporadic forms of the disorder are usually caused by aldosterone overproduction from a unilateral adrenocortical aldosterone-producing adenoma or from bilateral adrenocortical hyperplasia. The main knowledge-advances in disease pathophysiology focus on pathogenic germline and somatic variants that drive the excess aldosterone production. Less clear are the molecular and cellular mechanisms that lead to an increased mass of the adrenal cortex. However, the combined application of transcriptomics, metabolomics, and epigenetics has achieved substantial insight into these processes and uncovered the evolving complexity of disrupted cell growth mechanisms in primary aldosteronism. In this review, we summarize and discuss recent progress in our understanding of mechanisms of cell death, and proliferation in the pathophysiology of primary aldosteronism.

## Introduction

Primary aldosteronism (PA) is the most common form of secondary hypertension that accounts for 5 to 15% of patients with hypertension (1). The disorder encompasses a group of pathological conditions of the adrenal glands that trigger aldosterone overproduction associated with a higher risk of cardiovascular events (2). Thus, a prompt diagnosis of PA with accurate subtyping of unilateral from bilateral cases is essential to initiate specific treatment strategies (adrenalectomy or pharmacotherapy with a mineralocorticoid receptor antagonist) for optimal patient outcomes (1). Bilateral forms include familial forms of PA, which are rare monogenic forms of hypertension caused by germline pathogenic variants. To date, 4 genetically distinct forms of familial hyperaldosteronism have been identified (FH types 1 to 4), classified according to the affected gene (3, 4). Sporadic aldosterone-producing adenomas (APAs) carry somatic mutations in aldosterone-driver genes and in genes that drive the development of multiple adrenal tumors. In addition to genetic insights, a wealth of data has accumulated from transcriptomics and metabolomics analyses of aldosterone-producing lesions to gain a deeper understanding of PA pathophysiology.

## Genetics of primary aldosteronism

An extensive review on the genetics of PA has been published recently (4). In brief, FH type 1 is caused by a chimeric *CYP11B1*/*CYP11B2* gene composed of the adrenocorticotropic hormone (ACTH)-responsive promoter region of *CYP11B1* fused to the *CYP11B2* coding region (5). As a result, transcription of *CYP11B2* (aldosterone synthase) is under the regulatory control of ACTH instead of angiotensin II and potassium as in normal aldosterone physiology. The dysregulated aldosterone production in FH types 2, 3 and 4 is caused by variants in the ion channels encoded by *CLCN2* (CIC-2 chloride channel), *KCNJ5* (GIRK4 potassium channel), and *CACNA1H* (Cav3.2 calcium channel), respectively, that function in intracellular ion homeostasis. In addition, germline pathogenic variants in *CACNA1D* can cause a complex form of PA with seizures and neurologic abnormalities (PASNA) (**Table 1**).

**Table 1:** Effect Of Gene Variants In Primary Aldosteronism Driver Genes.

GENE	PROTEIN	MECHANISM IN PA PATHOPHYSIOLOGY	PA SUBTYPE			REFERENCE
**Ion Channels**						
*KCNJ5*	Potassium inwardly rectifying channel subfamily J member 5	Activation of Ca^2+^ signaling and dysregulated aldosterone production; deregulated cell growth	Somatic Germline	Unilateral Bilateral	APA FH-III	Choi M, 2011 (6)
*CACNA1D*	Calcium voltage-gated channel subunit alpha1 D	Activation of Ca^2+^ signaling and dysregulated aldosterone production	Somatic Somatic Germline	Unilateral Bilateral Bilateral	APA APM PASNA	Scholl UI, 2013 (7) Azizan EA, 2013 (8) Omata K, 2018 (9)
*CACNA1H*	Calcium voltage-gated channel subunit alpha1 H	Activation of Ca^2+^ signaling and dysregulated aldosterone production	Germline Somatic	Bilateral Unilateral	FH-IV APA	Scholl UI, 2015 (10) Daniil G, 2016 (11) Nanba K, 2020 (12)
*CLCN2*	Chloride voltage-gated channel 2	Activation of Ca^2+^ signaling and dysregulated aldosterone production	Germline Somatic	Bilateral Unilateral	FH-II APA	Scholl UI, 2018 (13) Fernandes-Rosa FL, 2018 (14) Dutta RK, 2019 (15)
**Ion Transporters**						
*ATP1A1*	ATPase Na^+^/K^+^ transporting subunit alpha 1	Activation of Ca^2+^ signaling and dysregulated aldosterone production; deregulated cell growth	Somatic Somatic	Unilateral	APA APM	Beuschlein F, 2013 (16) Azizan EA, 2013 (8)
*ATP2B3*	ATPase plasma membrane Ca^2+^ transporting 3	Intracellular acidification and dysregulated aldosterone production	Somatic Somatic	Unilateral	APA APM	Beuschlein F, 2013 (16)
**Cell Signaling Systems**						
*CTNNB1*	Catenin beta 1	Aldosterone overproduction; deregulated cell growth	Somatic	Unilateral	APA	Åkerström T, 2016 (17)
*GNA11*	G protein subunit alpha 11	Aldosterone overproduction; deregulated cell growth	Somatic	Unilateral APA (when coincident with *CTNNB1* variant)		Zhou J, 2021 (18)
*GNAQ*	G protein subunit alpha Q					
*GNAS*	GNAS complex locus	Deregulated cell growth	Somatic	Unilateral	APA	Nakajima Y, 2016 (19)
*PRKACA*	Protein kinase cAMP-activated catalytic subunit alpha	Deregulated cell growth	Somatic	Unilateral	APA	Rhayem Y, 2016 (20)
*ARMC5*	Armadillo repeat containing 5	Deregulated cell growth	Somatic	Unilateral	APA	Zilbermint M, 2015 (21)
**Cytochrome P450 Enzyme**						
*CYP11B1*/*B2*	Ectopically expressed aldosterone synthase	Dysregulated aldosterone production	Germline	Bilateral	FH-I	Lifton RP, 1992 (5)

APA, aldosterone-producing adenoma; APM, aldosterone-producing micronodule; FH, familial hyperaldosteronism; PA, primary aldosteronism; PASNA, PA, seizures, and neurologic abnormalities.

The landscape of somatic APA mutations overlaps with the germline pathogenic variants. Thus, *KCNJ5*, *CACNA1D*, *CACNA1H*, and *CLCN2* can carry somatic or germline mutations in PA. In APAs, this set of aldosterone-driver genes is complemented by somatic mutations in *ATP1A1* and *ATP2B3*, which encode the ion pumps Na^+^/K^+^-ATPase and Ca^2+^-ATPase, and in genes that are targeted in multiple adrenal tumors such as *CTNNB1* (β-catenin), and *GNA11*, *GNAQ*, and *GNAS*, encoding (guanine nucleotide-binding proteins), *PRKACA* (catalytic subunit of protein kinase A), and *ARMC5* (a member of the armadillo/β-catenin-like repeat family) (**Table 1**). The potential function of PA-related mutations in cell growth mechanisms is discussed below.

## Aldosterone-driver mutations and deregulated cell growth

The role of recurrent PA mutations in aldosterone overproduction is clearly demonstrated but less evident is their role in perturbing regulatory mechanisms of adrenal cell growth that lead to an increase of the adrenal cell mass and tumor development.

### GIRK4 potassium channel

**Figure 1:**
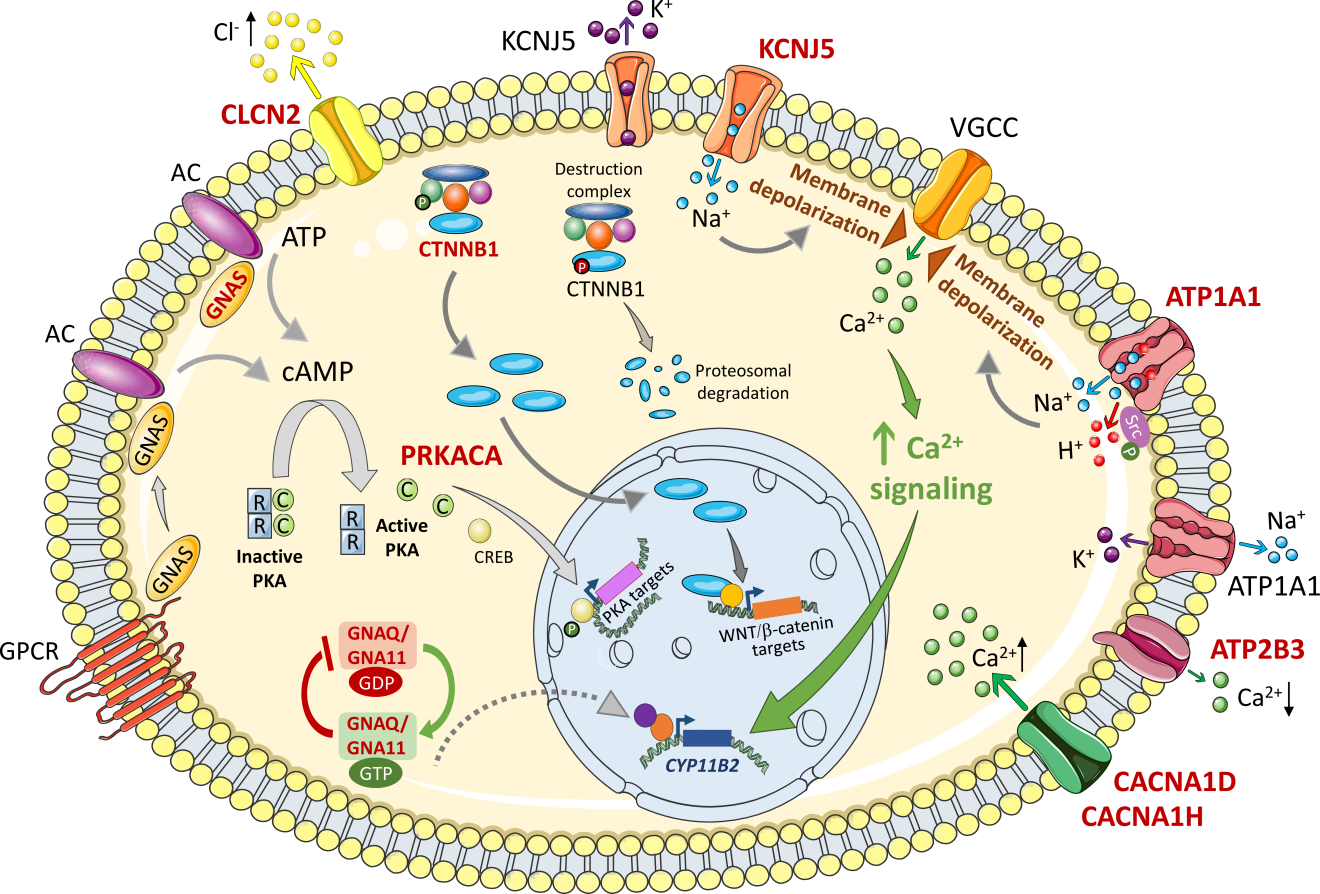
Mechanisms of deregulated cell growth of variants related to primary aldosteronism. The figure shows intracellular signaling pathways activated by APA somatic mutations implicated in cell growth mechanisms. Wild type and mutated forms are labeled in black and red respectively. Membrane depolarization triggered by Na+ or H+ influx causes opening of voltage gated ion channels (VGCC), Ca2+ influx and activation of Ca2+ signaling that activates CYP11B2 gene transcription to drive increased aldosterone biosynthesis and cell proliferation. ATP1A1 is also reported to activate cell proliferation by phosphorylated Src. Intracellular β-catenin concentrations are kept low under basal conditions by continuous proteosomal degradation. Activating mutations in exon 3 of the β-catenin gene bypass this control mechanism resulting in cytoplasmic β-catenin accumulation and nuclear translocation to activate specific gene transcription programs. In many adrenal tumours including some rare APA examples, cAMP/PKA signaling is activated by mutations in GNAS and PRKACA for activation of target genes. GNAQ and GNA11 mutations can cause upregulated aldosterone production *in vitro* and are associated with hyperplasia of glomerulosa cells adjacent to an APA. They are clinically silent *in vivo* except when concomitant with CTNNB1 (β-catenin) mutations. See text for details. Somatic mutations in CLCN2, CACNA1D, CACNA1H, and ATP2B3 have been described in APAs which result in increased Cl- efflux (CLCN2), increased Ca2+ influx (CACNA1D, CACNA1H), and decreased Ca2+ efflux (ATP2B3). AC, adenylyl cyclase; ATP, adenosine triphosphate; ATP1A1, ATPase Na+/K+ transporting subunit α1; ATP2B3, ATPase plasma membrane Ca2+ transporting 3; C, catalytic subunit; CACNA1D, calcium voltage-gated channel subunit alpha1 D; CACNA1H, calcium voltage-gated channel subunit alpha1 H; cAMP, cyclic adenosine monophosphate; Ca2+, calcium ions; CLCN2, chloride voltage-gated channel 2; CREB, cAMP response element-binding protein; CYP11B2, cytochrome P450 family 11 subfamily B member 2 (aldosterone synthase); GDP, guanosine diphosphate; GNA11, G protein subunit α11; GNAS, GNAS complex locus; GNAQ, G protein subunit αQ; GTP, guanosine triphosphate; GPCR, G protein coupled receptor; H +, hydrogen ions; K+, potassium ions; KCNJ5, potassium inwardly rectifying channel subfamily J member 5; Na+, sodium ions; PKA, protein kinase A; PRKACA, protein kinase cAMP-activated catalytic subunit α; R, regulatory subunit; VGCC, voltage-gated calcium channel; WNT, wingless-related integration site. Figure produced using Servier Medical Art (https://smart.servier.com/).

Choi et al. (6) identified somatic APA mutations and an inherited germline mutation in *KCNJ5* (encoding Gly151Arg and Leu168Arg somatic, and Thr158Ala germline variants in GIRK4, G-protein-gated inwardly rectifying K^+^ channel 4). This triggered a rapid evolution of research and the description of somatic mutations in other genes (7, 8, 12, 16, 17, 22–27) and the identification of additional familial forms of PA (10, 11, 13, 14). The *KCNJ5* variants result in single amino acid substitutions in or close to the selectivity filter of the encoded GIRK4 potassium channel. The channel properties are consequently altered causing loss of potassium selectivity and abnormal sodium influx (instead of exclusive K^+^ efflux) from glomerulosa cells. The resultant depolarization of zona glomerulosa cells causes opening of voltage-gated calcium channels, increased intracellular calcium concentrations and activation of calcium signal transduction and *CYP11B2* (aldosterone synthase) gene transcription and increased aldosterone production (6) (**Figure 1**). *KCNJ5* mutations are highly prevalent in APAs (3, 4), associated with larger adenoma size, female sex, and more severe aldosteronism (28). The application of genotype analysis to CYP11B2 (aldosterone synthase) immunopositive regions of paraffin-embedded adrenal sections combined with next generation sequencing (29) has resulted in an increased detection of somatic APA mutations such that prevalence rates are now described in around 90% of cases (22–25, 27, 30).

Germline *KCNJ5* mutations can cause FH type 3 which presents with a variable clinical phenotype that might be explained by the functional properties of the mutated GIRK4 potassium channel. This is supported by the report of 4 families with FH type 3 caused by either of 2 different *KCNJ5* mutations (31). Two families with GIRK4-Gly151Arg mutations presented with massive adrenocortical hyperplasia and severe aldosteronism requiring bilateral adrenalectomy. The other 2 families carried a Gly151Glu mutation associated with a milder PA phenotype without evident adrenal hyperplasia on imaging and easily controlled hypertension with pharmacotherapy. The Gly151Glu mutation caused higher sodium influx and greater consequent adrenal cell death than Gly151Arg accounting for the absence of structural changes at adrenal imaging in patients with the GIRK4-Gly151Glu germline variant (31). The cell toxicity of GIRK4 mutants can be circumvented by low levels of gene transcription and expression of the mutated GIRK4 channel (32).

### Sodium/potassium-transporting ATPase

The Na^+^/K^+^-ATPase pump couples the hydrolysis of an ATP molecule to the transport of 3 Na^+^ out and 2 K^+^ into the cell. Azizan et al. (8) and Beuschlein et al. (16), reported somatic gain-of-function mutations in *ATP1A1* (Na^+^/K^+^-ATPase alpha-1 subunit). *ATP1A1* variants are found in 5-17% of APAs (3) and many have been shown to severely impair K^+^ binding and ATPase activity conferring membrane depolarization in the cells which express them (8, 16, 33). Adrenal cells expressing *ATP1A1* mutations show normal intracellular Ca^2+^ concentrations suggesting that activated Ca^2+^ export pathways, *via* a Na^+^/Ca^2+^ exchanger or Ca^2+^-ATPase, or inactivation of Ca^2+^ channels might function as compensatory mechanisms (34). However, adrenal cells expressing *ATP1A1* mutants exhibit abnormal H^+^ leak currents that cause intracellular acidification that can lead to increased expression of *CYP11B2* (aldosterone synthase) (34) (**Figure 1**).

Na^+^/K^+^-ATPase can display a dual function not only as an ion pump but also as a signaling complex relaying extracellular signals to intracellular compartments (35–37). For example, ouabain- the cardiotonic steroid- can bind to Na^+^/K^+^-ATPase which in turn interacts, *via* its alpha-1 subunit, with the Src proto-oncogene tyrosine kinase. Subsequent Src activation by phosphorylation then enhances downstream signaling pathways (37). Human adrenal cells expressing Na^+^/K^+^-ATPase-p.Leu104Arg (a recurrent somatic APA mutation) show increased levels of Src phosphorylation and enhanced cell proliferation implicating a potential novel mechanism of the Na^+^/K^+^-ATPase-Src functional complex in the progression and development of APAs with an *ATP1A1* mutation (38) (**Figure 1**).

### Calcium-transporting ATPase, Cav1.3 and Cav3.2 calcium channels, and the CIC-2 chloride channel

As mentioned above, somatic APA mutations have been identified in other genes involved in intracellular ion homeostasis (*ATP2B3*, *CACNA1D*, *CACNA1H*, and *CLCN2*). However, there is no experimental evidence to demonstrate a potential role for mutations in these genes in cell proliferation or cell death. *ATP2B3* encodes the Ca^2+^-ATPase which pumps intracellular Ca^2+^ across the plasma membrane to the extracellular environment. The Ca^2+^-ATPase mutations in PA interfere with Ca^2+^ ion binding resulting in decreased export and increased Ca^2+^ influx.

Heterozygous somatic mutations in the L-type voltage gated Ca^2+^ channel Cav1.3 (encoded by *CACNA1D*) are frequently described in aldosterone-producing lesions associated with PA (in APAs and in aldosterone-producing micronodules [APMs]) (7–9) and more rarely in the T-type Ca^2+^ channel Cav3.2 (encoded by *CACNA1H*) (12). PA-driver mutations in *CACNA1D* and *CACNA1H* confer a gain-of-function and result in increased Ca^2+^ influx. Somatic APA mutations in C*LCN2* encoding the CIC-2 chloride channel have been described in just a few cases (15).

## Driver mutations of tumorigenesis in primary aldosteronism

Somatic mutations that constitutively activate WNT/β-catenin (*CTNNB1*), G-protein (*GNAS*, *GNAQ*, *GNA11*), and cAMP/protein kinase A (PKA) (*GNAS*, *PRKACA*) signaling can drive unrestrained cell proliferation and survival in diverse tumors, including adrenal tumors. Activating mutations in β-catenin (encoded by *CTNNB1*) that cause WNT-independent signaling are widely reported in APAs (17, 39). Constitutive cAMP/PKA signaling due to *PRKACA* and *GNAS* activating mutations has been reported in a limited number of APAs (19, 20). Mutations in G protein α subunits, particularly those encoded by *GNAS* and *GNAQ*/*GNA11*, have been reported in many tumours including a wide variety of endocrine tumors (40, 41).

### Activation of WNT/β-catenin signaling

The regulated destruction of the transcriptional co-activator β-catenin is central to the WNT-mediated β-catenin signaling cascade. This process involves a multi-subunit destruction complex, comprising AXIN (axis inhibitor protein), APC (tumor suppressor adenomatous polyposis coli), and GSK-3β (glycogen synthase kinase-3), which phosphorylates β-catenin for ubiquitylation and proteasomal degradation. In contrast, WNT-mediated cell stimulation disrupts β-catenin degradation causing its accumulation and subsequent nuclear translocation to co-activate the transcription of WNT target genes (42).

Mutations that drive continuous activation of β-catenin signaling are widely reported in human cancers and in several adrenal tumors (43). Consistent with the proliferative advantage of adrenal cells with activating *CTNNB1* mutations, transgenic mice with constitutively active β-catenin display adrenal cell hyperproliferation and adrenal hyperplasia (44) (**Figure 1**). Many studies implicate disrupted WNT/β-catenin signaling as a key mechanism in APA pathogenesis. This pathway is constitutively activated in 70% APAs, an observation related to the decreased expression of an endogenous inhibitor of the WNT/β-catenin signaling called SFRP2 (secreted frizzled related protein 2) (45). Activating somatic *CTNNB1* mutations located in exon 3 have been identified in 1% to 5% of APAs (17, 18, 46). Of note, 16 of 27 (59%) APAs with a *CTNNB1* activating mutation carry a concurrent G protein α subunit substitution mutation of a highly conserved glutamine residue (p.Gln209) in GNAQ or GNA11 (18) (discussed in more detail below). Overall, *CTNNB1* mutations are found in only a small proportion of APAs with β-catenin cytoplasmic accumulation and nuclear translocation (17, 45, 47) thereby suggesting a role for other factors, other than mutations, in the disruption of WNT/β-catenin signaling in the pathogenesis of APAs (48).

### Inactivation of the ARMC5 tumour suppressor protein

Germline and somatic inactivating mutations in the putative tumor-suppressor gene *ARMC5* (armadillo repeat containing 5)- a member of the armadillo/β-catenin-like repeat superfamily- can cause primary bilateral macronodular adrenal hyperplasia (a rare form of primary adrenal Cushing syndrome) (49, 50). ARMC5 has multiple binding partners (51) and may function in PKA, and the WNT/β-catenin signaling pathways (52). Armc5 plays a vital role in early mouse embryonic development, and the T-cell immune response. In addition, the development of adrenal hyperplasia with increasing age in Armc5 knock out mice illustrates the key role of Armc5 in the adrenal (51, 52).

Zilbermint et al. (21) identified germline *ARMC5* variants in 22 of 56 (39.3%) patients with PA. These included variants predicted to be damaging by in silico analysis in 6 of 56 (10.7%) patients. All patients carrying a damaging *ARMC5* variant were African Americans suggesting that ARMC5 pathogenic variants might contribute to the known increased predisposition of this population to low renin hypertension (21). In contrast, *ARMC5* mutations in the coding sequence or intron-to-exon boundaries in a cohort of 37 Caucasian patients with PA due to bilateral adrenal lesions were not identified (53).

### Activation of G protein signaling

Abnormal expression and activation of G proteins (guanine nucleotide-binding proteins) are frequent features of tumorigenesis (40) with alterations in the G protein α subunits GNAS, GNAQ, and GNA11 commonly detected (41). G protein α subunits are components of the heterotrimeric G protein complex (comprising a Gα, Gβ, and Gγ subunit) which mediates G protein coupled receptor signal transduction. Signal activation is achieved by GDP for GTP exchange on the Gα subunit, hydrolysis of GTP to GDP terminates the signal. Recurrent mutations in GNAS, GNAQ and GNA11 disturb GTPase activity thus driving constitutively active and prolonged signaling (**Figure 1**).

GNAS is one of the most recurrently mutated G proteins in human cancer (40). GNAS mutations frequently alter either a p.Arg201 or a p.Gln227 residue, which are required for GTPase activity. GNAS mutations are found in a substantial proportion of cortisol-producing adenomas associated with subclinical mild autonomous cortisol excess (5 of 7, 71.4%) (54). Only a few APA with GNAS mutations (GNAS p.Arg201Cys) have been described and these were in cases of PA associated with autonomous cortisol secretion and activation of cAMP/PKA (protein kinase A) signaling (19).

Hotspot somatic mutations in the G protein α subunits GNAQ and GNA11 are found in 295 of 8778 (3.4%) and 155 of 6237 (2.5%) of human tumors, respectively (40, 55). Most are substitution mutations of residues p.Arg183 and p.Gln209 (homologous to p.Arg201 and p.Gln227 in GNAS) that impair GTP hydrolysis. However, mutations of p.Arg183 in GNAQ and GNA11 maintain some sensitivity to regulators of G protein signal termination and are less damaging variants.

As mentioned above, Zhou et al. (18) reported a high prevalence of APA *GNA11* or *GNAQ* mutations coexisting with *CTNNB1* mutations. The *GNA11* or *GNAQ* mutations caused substitution of the conserved p.Gln209 residue for either a His, Pro, or Leu amino acid, thereby driving their constitutive activation. Transfection of primary adrenocortical cells with *CTNNB1* and *GNA11* mutants, individually or combined, caused increased aldosterone production. Solitary *GNA11* mutations were also identified in the hyperplastic zona glomerulosa layer of the adrenal cortex adjacent to APAs with *GNA11* and *CTNNB1* mutations (18). This supports the 2-hit model of tumorigenesis in APA development (56) in which a first event stimulates adrenocortical cell proliferation, and a second event (a somatic aldosterone-driver mutation) drives autonomous aldosterone production (4, 57, 58).

### Activation of cAMP/protein kinase A signaling

Cyclic adenosine monophosphate (cAMP) regulates multiple cellular functions in most cell types *via* the control of target gene transcription mainly mediated by protein kinase A (PKA). Adenylyl cyclase is activated by GNAS binding thus enabling the conversion of adenosine triphosphate (ATP) to cAMP, which in turn activates PKA. The PKA heterotetramer comprises two regulatory subunits (PRKAR1A) and two catalytic subunits (PRKACA). The regulatory subunits maintain the inactivity of the catalytic subunits in the tetrameric complex. cAMP binding to each regulatory subunit causes their dissociation and activation (59). PKA regulates transcription by direct phosphorylation of transcription factors such as CREB (cAMP-response element-binding protein), and CREM (cAMP-responsive modulator) so they can bind to cAMP-response elements in target genes.

Constitutive activation of cAMP/PKA signaling due to *GNAS* or *PRKACA* mutations is linked to the formation of many tumors including endocrine tumors (60, 61). The rare examples of APA *GNAS* mutations are discussed above (under G protein signaling). Somatic *PRKACA* mutations, encoding a p.Leu206Arg substitution in the catalytic PKA subunit, have been identified in a substantial proportion of cortisol-producing adenomas (54, 60, 62, 63). PRKACA Leu206 directly interacts with the PKA regulatory subunit. The PRKACA p.Leu206Arg mutation disrupts this interaction causing PRKACA constitutive activation with consequent increased phosphorylation of downstream targets (60). Expression of PRKACA p.Leu206Arg *in vitro* causes increased basal PKA activity and cAMP signaling (60, 62), which can account for the dysregulated cortisol production and cell proliferation of these tumors. In PA, PRKACA p.Leu206Arg mutations are rare (20, 60) and seem to occur in some patients with cortisol co-secretion. In addition, the adenoma with the PRKACA p.Leu206Arg mutation can be negative for CYP11B2 (aldosterone synthase) immunostaining (64, 65) indicating the likely function of this mutation in tumor formation.

## Transcriptomics of aldosterone-producing adenomas

APA transcriptome analyses show considerable heterogeneity in methodology (different reference tissues, different gene expression platforms). Despite this, many studies report consistent findings, which have helped define the panorama of transcriptome changes in APA formation and have highlighted genes and signaling pathways that function in dysregulated aldosterone production (**Table 2**). Below we focus on differentially expressed genes in APAs which are implicated in mechanisms of cell death and proliferation. The application of transcriptomics to identify mechanisms of aldosterone overproduction are described elsewhere (66, 67).

### Mechanisms of cell death

Early reports relied on microarray gene expression studies. Transcriptome comparison of 8 APAs with those of 3 normal adrenals identified a range of differentially expressed genes which included *TDGF1* (teratocarcinoma-derived growth factor 1)- also reported as upregulated by a SAGE (serial analysis of gene expression) study (68)- and *VSNL1* (visinin-like 1) encoding a calcium binding protein (69, 70). Overexpression of *TDGF1* and *VSNL1* in human adrenocortical cells *in vitro* suggested that each gene played a role in cell survival by protection against cell death by apoptosis (69, 70). The nuclear transcription factor and WNT/β-catenin target *AFF3*, also protects adrenal cells from apoptosis and is a positive regulator of adrenal cell proliferation (71). mRNA-seq analysis of 15 APAs suggested a role for *AFF3* in APA pathophysiology by demonstrating upregulated *AFF3* gene expression in *CTNNB1* mutated APAs versus those without a *CTNNB1* mutation (72) (**Table 2**).

**Table 2:** Potential function of differentially expressed genes and Mirnas in Aldosterone-Producing Lesions.

GENE or miRNA	PROTEIN	TRANSCRIPTOME COMPARISON		FUNCTIONAL EFFECT IN ADRENOCORTICAL CELLS	GENE EXPRESSION PLATFORM	VALIDATION	REFERENCE
		SAMPLE TISSUE	REFERENCE TISSUE				
**Upregulated gene expression**							
*AFF3*	AF4/FMR2 Family Member 3	APA with *CTNNB1* variant (*n*=3)	APA without *CTNNB1* variant (*n*=12)	Negative regulation of cell death by apoptosis. Positive regulator of cell proliferation.	RNAseq	Yes (49 APAs)	Backman S, 2019 (72)
*BEX1*	Brain expressed X-linked 1	APA < 10 mm diam. (*n*=12)	APA ≥ 30 mm diam. (*n*=9)	Negative regulation of cell death by ferroptosis.	RNAseq	Yes (71 APAs)	Yang Y, 2021 (73)
		APA with *CACNA1D* or *ATP1A1* variant (*n*=5)	APA with *KCNJ5* variant (*n*=8)		Microarray	No	Azizan EA, 2013 (8)
		APM (*n*=4)	Paired adjacent zG (*n*=4)		Microarray	No	Nishimoto K, 2015 (74) Yang Y, 2021 (73)
*NEFM*	Neurofilament medium chain	APA without *KCNJ5* variant (with compact eosinophilic cells) (*n*=7)	APA with *KCNJ5* variant (with clear cells) (*n*=7)	Negative regulator of cell proliferation. Suppressor of aldosterone production	Microarray	Yes	Zhou J, 2016 (75) Maniero C, 2017 (76)
*SHH*	Sonic hedgehog	APA (*n*=12)	Normal adrenals (*n*=6)	Positive regulator of cell proliferation.	*In situ* hybridization	No	Boulkroun S, 2011 (47) Werminghaus P, 2014 (77) Gomes DC, 2014 (78)
*TDGF1*	Teratocarcinoma-derived growth factor 1	APA (*n*=8)	Normal adrenals (*n*=3)	Negative regulation of cell death by apoptosis. Stimulation aldosterone production.	Microarray	Yes (19 APAs versus 10 normal adrenals)	Williams 2010 (69)
		APA (*n*=1)	Paired adjacent cortex (*n*=1)		SAGE	–	Assié G, 2005 (68)
*VSNL1*	Visinin like 1	APA (*n*=8)	Normal adrenals (*n*=3)	Negative regulation of cell death by apoptosis. Stimulation aldosterone production.	Microarray	Yes (19 APAs versus 10 normal adrenals)	Williams TA, 2010 (69) Williams TA, 2012 (70)
*YPEL4*	Yippee Like 4	APA (*n*=39)	Nonfunctional adrenoma (*n*=12)	Positive regulator of cell proliferation. Stimulation of aldosterone production. Positive correlation with APA diameter.	qRT-PCR	–	Oki K, 2016 (79)
**Downregulated gene expression**							
*LGR5*	Leucine rich repeat containing G protein-coupled receptor 5	APA (*n*=14)	Paired adjacent zG (*n*=14)	Positive regulation of apoptosis. Negative regulator of cell proliferation. Suppresses aldosterone production.	Microarray	Yes	Shaikh LH, 2015 (80) Zhou J, 2016 (75)
**Downregulated miRNA expression**							
miR-193a-3p	–	APA (*n*=15)	Paired adjacent cortex (*n*=15)	Negative regulation of cell proliferation. Suppresses aldosterone production	qRT-PCR	–	Zhang G, 2018 (81)
miR-203	–	APA (*n*=10)	Paired adjacent cortex (*n*=10)	Negative regulation of cell proliferation. Negative correlation with APA diameter	Microarray	Yes (40 APAs versus 40 paired adjacent cortex)	Peng KY, 2018 (82)
miR-375	–	APA (*n*=6)	Normal adrenals (*n*=4)	Negative regulation of cell proliferation. Negative correlation with APA diameter.	Microarray	Yes (88 APAs, 16 normal adrenals)	He J, 2015 (83)

Transcriptome analysis of APAs and paired adjacent zona glomerulosa highlighted a potential role for oxidative stress in APA pathogenesis (75). The top canonical biological pathway associated with the differentially expressed genes was NRF2 (nuclear factor erythroid 2–related factor 2)-mediated oxidative stress, which is a critical cellular mechanism to maintain intracellular redox homeostasis and limit oxidative damage (84). An imbalance in redox-based metabolic processes can result in inappropriate production of reactive oxygen species (ROS) and oxidative damage to membrane lipids. If cellular antioxidant systems are unable to inhibit oxidative damage, a chain reaction of lipid peroxidation occurs, and cells commit to a form of regulated cell death called ferroptosis (85). GPX4 (glutathione peroxidase 4) is an essential antioxidant peroxidase which catalyzes the reduction of lipid hydroperoxides to protect cells from ferroptosis. Adrenocortical cells are particularly sensitive to inducers of ferroptosis which act *via* GPX4 inhibition (86). Functional enrichment analysis of differentially expressed genes in APAs compared with adjacent zona glomerulosa identified a ferroptosis-related gene set (87). The upregulated genes included *SCD* and *GCLC*, which encode key enzymes with a ferroptosis protective function in the catalysis of monounsaturated fatty acids and glutathione biosynthesis.

**Figure 2:**
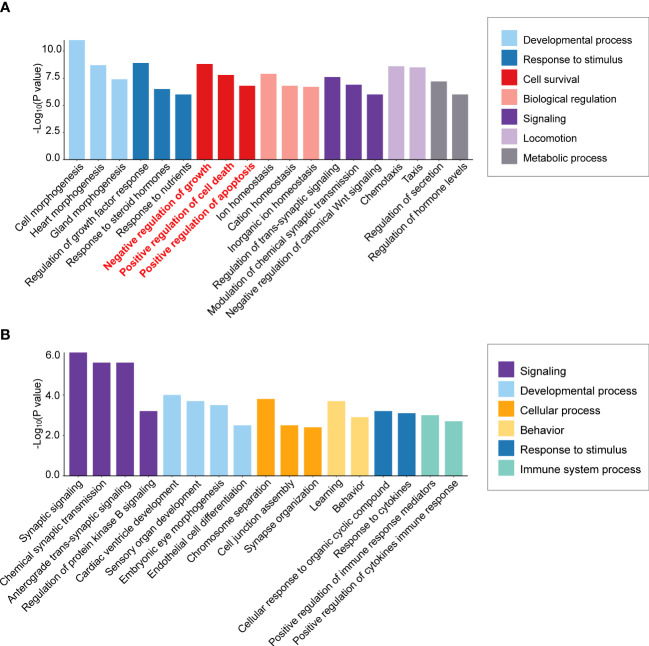
Differential enrichment of cell survival pathways in aldosterone-producing adenomas according to *KCNJ5* (GIRK4) mutation status. Biological process enrichment analysis was performed of significantly differentially expressed genes in macro versus micro APAs (≥30 mm versus ≤10 mm adenoma diameter) without a KCNJ5 mutation **(A)** and with a KCNJ5 mutation **(B)** using Metascape (http://metascape.org/accessed on 26 February 2022) analysis of publicly available dataset (https://github.com/MedIVLMUMunich/MacroMicroAPA_RNAseq). Enrichment visualization was performed using R package ggplot2 (v3.3.5).

To detect mechanisms of APA tumorigenesis, Yang et al. (73) compared APA transcriptomes of highly diverse sizes (9 macro APAs with adenoma diameter ≥30 mm versus 12 micro APAs ≤10 mm). Over-representation analysis of the transcriptome dataset from Yang et al. (73) illustrates enrichment of cell survival pathways (negative regulation of cell growth, positive regulation of cell death, and positive regulation of apoptotic processes) in the *KCNJ5* mutation-negative macro versus micro APAs but not in *KCNJ5*-mutated APAs (**Figures 2A**, **B**). Thus, activation of gene expression programs with a negative impact on cell survival can limit the size of APAs without a *KCNJ5* mutation. In contrast, this is not a feature of APAs with a *KCNJ5* mutation which are characterized by their larger tumor size (28).

Validation of expression levels of genes associated with processes of cell death and cell proliferation in an expanded sample set of 71 APAs identified a subset of genes with transcription profiles strongly correlated with adenoma diameter in *KCNJ5*-mutation negative APAs (73). These included *BEX1* (brain expressed X-linked 1) which was inversely correlated with APA diameter and was upregulated in APAs relative to paired adjacent cortex. A previous study (8) reported upregulated *BEX1* gene expression in APAs with a *CACNA1D* or *ATP1A1* mutation (that tend to be small adenomas) compared with the larger *KCNJ5*-mutated APAs (8, 28). Flow cytometry analyses of human adrenocortical cells with stable BEX1 overexpression demonstrated its role in protection from cell death by ferroptosis, rather than by apoptosis or by interference with cell cycle progression (73). The relatively high *BEX1* gene expression in smaller versus larger APAs and in aldosterone-producing micronodules (APMs) compared with adjacent zona glomerulosa cells (73, 74) implicates a role for *BEX1* in the promotion of cell survival in the initiation of adenoma formation.

### Mechanisms of cell proliferation

Genes involved in calcium signaling were first highlighted by some of the first APA gene expression studies (88), consistent with the later discovery of activation of the calcium signaling system by aldosterone-driver mutations (89). Signaling cascades initiated by intracellular Ca^2+^ are ubiquitously employed for the regulation of cell proliferation. This is achieved at several levels including by the promotion of resting G0 cell entry to the cell cycle, activation of the initiation of DNA synthesis at the G1 to S phase transition, and by stimulation of mitosis (90). Thus, in addition to a role in aldosterone production, genes which function in calcium signaling might also mediate cell proliferation, such as those with a calcium binding function such as *VSNL1* (visinin-like), *CALN1* (calneuron 1) and *CLGN* (calmegin), which are all significantly upregulated in APAs (70, 91, 92).

The *YPEL* (yippee-like) gene family encodes proteins with putative zinc-finger-like metal-binding domains (yippee domains). The encoded proteins localize to the centrosome and nucleolus and have been proposed to regulate cell division and proliferation (93). Transcriptome analysis of freshly isolated rat adrenal zona glomerulosa cells treated with angiotensin II (100 nM) or potassium (16 mM KCl) identified a set of genes, comprising *YPEL4*, that might function in aldosterone production (94). The upregulation of *YPEL4* in response to angiotensin II or K^+^ stimulation in rat adrenal cells was successively observed in human adrenocortical cells which caused increased adrenal cell proliferation (79). A role for *YEPL4* in adrenal cell proliferation in APAs was also suggested by the slight but significant positive correlation of *YPEL4* gene expression levels with APA diameter (79) (**Table 2**).

In a microarray study of 14 APAs, *LGR5* (leucine rich repeat containing G protein-coupled receptor 5) was downregulated in APA compared with adjacent zona glomerulosa (75, 80). LGR5 overexpression in human adrenal cells caused increased apoptosis, a reduction of proliferation, and decreased aldosterone production (80). Moreover, *NEFM* (neurofilament medium chain) was upregulated in APAs without a *KCNJ5* mutation relative to those with a *KCNJ5* mutation (75) and transfection of mutated *KCNJ5* into adrenal cells significantly decreased *NEFM* gene expression levels (60). Consistently, *NEFM* gene silencing resulted in increased adrenal cell proliferation and amplified aldosterone production (Table 2).

A large transcriptome analysis of APAs identified the potential role of the retinoic acid receptor alpha (RARα) in adrenal cell proliferation. Inactivation of *Rarα* in transgenic mice caused an inhibition of non-canonical Wnt signaling and increased proliferation in male mice (95). The study suggests that RARα might function in the structural maintenance of the adrenal cortex and that disrupted RARα signaling could be factor which contributes to APA pathogenesis.

## *In situ* metabolomics of aldosterone-producing adenomas and micronodules by mass spectrometry imaging

*In situ* matrix-assisted laser desorption/ionization mass spectrometry imaging (MALDI-MSI) is a novel technique that can simultaneously measure up to thousands of molecules in a single tissue section. The detected molecules encompass metabolites, lipids, glycans, peptides and proteins, as well as drugs and their metabolites (96). The approach allows co-integration of the detected molecules with conventional hematoxylin and eosin staining or immunohistochemistry and thus enables the spatial visualization of metabolite distribution, and other molecules including hormones, in the context of morphology or protein expression.

According to classical adrenal morphology, the adrenal comprises 3 distinct concentric zones of the cortex (glomerulosa, fasciculata, and reticularis) and the adrenal medulla. Using MALDI-Fourier transform-ion cyclotron resonance-MSI (MALDI-FT-ICR-MSI) applied to fresh frozen normal human adrenal sections, Sun et al. (97) determined a new molecular definition of adrenal gland anatomy. The study established the unexpected complexity of the adrenal by visualization of 10 clearly distinct molecular zones (6 cortical and 4 medullary substructures). The functional and physiological relevance of this multi-layered molecular anatomy remains to be determined but this technique has been extensively applied to extend our knowledge of adrenal pathology (98–100).

In addition to fresh frozen tissue sections, MALDI-FT-ICR MSI can reliably be performed on formalin-fixed paraffin embedded (FFPE) archived tissue samples, including individual biopsy specimens, and tissue microarrays comprising thousands of tissue cores. Comparison of mass spectrometry peaks from tissue specimens that had been fresh frozen or paraffin-embedded, revealed that similar metabolites were detected, with a 72% overlap, of comparable peak intensities, particularly for non-lipid low molecular weight metabolites (101).

MALDI-FT-ICR MSI analysis of a tissue microarray representing 132 APAs with genotype data showed that samples did not cluster according to genotype in the total dataset. However, restricting analysis to adenomas with either a *KCNJ5* or a *CACNA1D* mutation classified these 2 genotype groups with significant differences of 137 metabolites (98). Biological pathway analysis revealed enrichment of purine metabolism with increased purine synthesis in *KCNJ5*-mutated APAs, which are generally larger, than those with a *CACNA1D* mutation. Thus, the increased purine synthesis might conceivably result from cell cycle promotion and enhanced cell proliferation (98).

**Figure 3:**
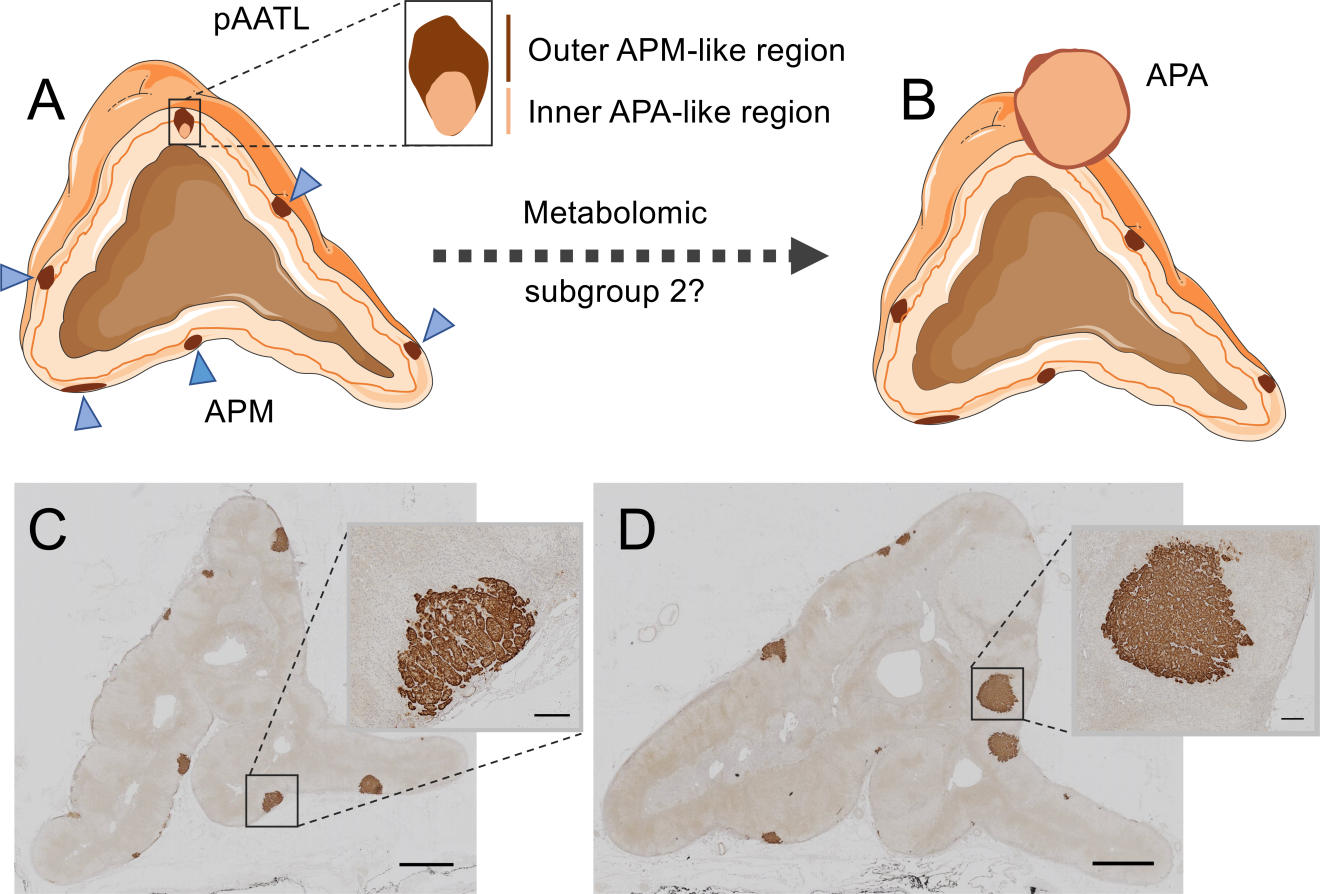
Hybrid lesions in the transition of aldosterone-producing micronodules to adenomas. The pAATL is a lesion composed of an outer APM-like region (with positive immunostaining for CYP11B2 but negative for CYP11B1) and an inner APA-like region (with positive immunostaining for both CYP11B2 and CYP11B1) that can have a different mutation status for PA-driver genes **(A)**. Thus, pAATLs have been proposed as hybrid APM-APA lesions and might represent intermediary lesions in the transit of APMs to APAs. Subgroups of APMs can be differentiated by their highly divergent metabolic profiles. A subset of APMs (metabolic subgroup 2) display a metabolic signature like that of APAs and might suggest progression to APAs **(B)**. CYP11B2 (aldosterone synthase) immunostaining of an adrenal surgically removed from a patient with PA **(C, D)**. The adrenal in Panel C shows multiple APMs (magnified in inset), a different sample block of the same adrenal in Panel D shows formation of aldosterone-producing nodules (magnified in inset). Bars = 2mm (main image) or 200 μm (inset). Blue triangles indicate APMs in Panel **(A)** APA, aldosterone-producing adenoma; APM, aldosterone-producing micronodule; pAATL, possible APM-to-APA transitional lesion; CYP11B1, 11β-hydroxylase; CYP11B2, aldosterone synthase. Figure produced using Servier Medical Art (https://smart.servier.com/).

Sugiura et al. (99) used MALDI-MSI with chemical derivatization to visualize specific steroids in fresh frozen adrenal tissue sections. The study generally confirmed the production of aldosterone in areas of CYP11B2 immunostaining in APAs and in aldosterone-producing micronodules (APMs) (99). APMs are small (<10 mm) CYP11B2-positive adrenal lesions located under the adrenal capsule (102, 103). They frequently carry somatic mutations in *CACNA1D*, *ATP2B3* and *ATP1A1* corresponding to those found in APAs (104). In contrast, *KCNJ5* mutations are largely absent in APMs, despite their high frequency in APAs (74). A small number of adrenal lesions with hybrid immunohistological features composed of an outer APM-like part, characterized by CYP11B2 but not CYP11B1 immunostaining, and an inner APA-like part, with both CYP11B2 and CYP11B1 immunostaining have been described (105). The occurrence of mutations common to both APMs and APAs and the observation of hybrid lesions led to the proposal that APMs might be precursors of APAs in some cases (105, 106) (**Figure 3**).

MALDI-FT-ICR MSI was used to determine the metabolomic phenotypes of 27 APMs and 6 APAs from surgically resected adrenals from patients with PA (100). The study established 2 subgroups of APMs (subgroups 1 and 2) with distinct metabolic phenotypes. The pattern of metabolites in APM subgroup 1 (20 of 27 APMs) was clearly separated from that of both subgroup 2 (7 of 27 APMs) and APAs. In contrast, the metabolic phenotype of APMs in subgroup 2 closely resembled that of APAs and displayed enrichment of biological pathways supporting cell proliferation and potentially tumour progression with increased purine synthesis characterizing APM subgroup 2 (100). Mutational status did not appear to account for the difference in metabolic signatures between the 2 APM subgroups and they were indistinguishable by immunohistology. Despite the apparent absence of hybrid lesions, these observations support the hypothesis of the “transitional lesion” in which a subgroup of APMs progress to APAs (100, 105) (**Figure 3**). Such a model in which an APM can transit to form an APA is an alternative to the 2-hit hypothesis of APA development (57) in which an initial event stimulates cell proliferation [such as a genetic variant (107) or circulating factor (108, 109)] and a subsequent somatic mutation in a stimulated cell, drives the aldosterone overproduction.

## Emerging role of epigenetics and micro RNAs

Increasing numbers of studies report the disruption of epigenetic and post-transcriptional mechanisms, notably by DNA methylation and microRNAs (miRNA/miR), as mechanisms which might facilitate APA pathogenesis.

### DNA methylation in the pathophysiology of aldosterone-producing adenomas

Several studies highlight deregulated DNA methylation as an epigenetic mechanism in APA pathogenesis. Gene expression is typically repressed by cytosine methylation of CpG (5’-cytosine-guanine-3’ dinucleotide) islands in target gene promoter regions. APAs display a distinct pattern of methylation (hypomethylated status) compared with their adjacent adrenal cortex and non-functioning adrenal adenomas (110, 111). *CYP11B2* was hypomethylated and upregulated, a process which was bypassed in the presence of either APA *KCNJ5* or *ATP1A1* mutations (110–112). Murakami et al. (113) integrated a genome-wide methylome analysis with transcriptome data and demonstrated that many genes involved in tumorigenesis (*HOX* family genes, *PRRX1*, *RAB38*, *FAP*, *GCNT2*, and *ASB4*) and steroidogenesis (*CYP11B2*, *MC2R*, and *HPX*) were hypomethylated and upregulated in APAs compared with adjacent cortex.

### Micro RNAs in the pathophysiology of aldosterone-producing adenomas

MicroRNAs (miRs, miRNAs) are small molecules, 18-22 nucleotides in length, that regulate post-transcriptional gene expression levels usually *via* downregulating the expression of specific genes by binding to the 3´untranslated regions of their corresponding mRNAs (114, 115). miRNA expression profiling can characterize diseased states and, as such, might be promising as a diagnostic tool (114). In PA, changes in circulating plasma miRNA expression levels have been reported in patients according to subtype with significant levels of miR-30e-5p, miR-30d-5p, and miR-7-5p overexpression in patients with bilateral adrenal hyperplasia versus those with a unilateral APA (116, 117).

In addition to circulating miRNAs, several studies have reported downregulated miRNAs in APA tissue samples compared with the adjacent adrenal cortex or relative to other resected adrenal specimens (**Table 2**). Downregulated miR-375 expression was reported in APA relative to both unilateral adrenal hyperplasia and normal adrenals. A role for miR-375 in tumorigenesis is supported by the reduction in adrenal cell viability in response to miR-375 overexpression *in vitro*. In addition, miR-375 expression levels are negatively correlated with APA diameter and are also downregulated in other tumors (82, 83). The putative tumorigenic function of miR-375 might be mediated by decreased expression of the target gene *MTDH* (metadherin) and subsequent suppression of Akt signalling (83). Peng et al. identified miRNA-203 as a candidate functional miRNA in APA pathophysiology from microarray-based expression analysis of 10 APA tissues and paired adjacent adrenal cortex (82). Treatment of human adrenal cells with miR-203 inhibitors caused increased aldosterone production and cell proliferation. Moreover, miR-203 mimics resulted in decreased adrenal cell proliferation as well as aldosterone hypersecretion from primary cell cultures derived from APA tissue. *WNT5A* was identified as a direct target of miR-203 implicating WNT5A/β-catenin signaling in mediating the observed functional effects (82).

## Perspectives

Diverse mechanisms alter the balance between adrenal cell death and proliferation to favor APA formation and development. These processes can vary according to genotype. Unrestrained proliferation of adrenal cells carrying a *KCNJ5* mutation contrasts with specific gene expression programs elicited in *KCNJ5* mutation negative APAs which influence adrenal cell viability and potentially regulate tumor size. The application of advanced omics technologies has tremendous potential to advance our understanding of the underlying biology of APA tumorigenesis. Accordingly, single-cell and spatially resolved transcriptomics will provide a detailed cellular atlas of PA adrenals and define gene expression profiles and genotype in selected cell populations. Integration of spatial transcriptomics with metabolic phenotyping using *in situ* mass spectrometry imaging will achieve a higher-level definition of biological pathways in cell subpopulations and elucidate the fundamental pathological processes in APA tumor cells.

## Author contributions

All authors listed have made a substantial, direct, and intellectual contribution to the work and approved it for publication.

## Funding

The Deutsche Forschungsgemeinschaft (DFG, German Research Foundation) supports TW and MR (project number 314061271 - TRR 205, and project number 444776998 [WI 5359/2-1 and RE 752/31-1]). The Else Kröner-Fresenius Stiftung (2012_A103, 2015_A228, and 2019_A104; Else-Kröner Hyperaldosteronismus- German Conn Registry) also supports the work of MR.

## Acknowledgments

We gratefully acknowledge the excellent assistance of Isabella-Sabrina Kinker with Figures.

## Conflict of interest

The authors declare that the research was conducted in the absence of any commercial or financial relationships that could be construed as a potential conflict of interest.

## Publisher’s note

All claims expressed in this article are solely those of the authors and do not necessarily represent those of their affiliated organizations, or those of the publisher, the editors and the reviewers. Any product that may be evaluated in this article, or claim that may be made by its manufacturer, is not guaranteed or endorsed by the publisher.
